# Unmet ADL/IADL Needs among Veterans with Dementia: An Analysis of the HERO CARE Multi‐site Survey

**DOI:** 10.1002/alz.095644

**Published:** 2025-01-09

**Authors:** Marianne E Desir, Richard A Munoz, Jared Hansen, Fei Tang, Stuti Dang

**Affiliations:** ^1^ Miami Veterans Affairs Healthcare System, Miami, FL USA; ^2^ Geriatric Research, Education and Clinical Center, Miami, FL USA; ^3^ South Florida VA Foundation for Research and Education, Miami, FL USA; ^4^ VA Salt Lake City Health Care, Salt Lake City, UT USA; ^5^ Miami VA Healthcare System GRECC, Miami, FL USA

## Abstract

**Background:**

Receiving sufficient assistance with ADLs/IADLs can support dementia patients’ physical and psychosocial health, as well as aging in place. The Department of Veterans Affairs has prioritized supporting Veterans in aging at place a home when this is their preference. We aimed in the current study to analyze if there are demographic characteristics and/or comorbidities of Veterans with dementia that are predictive of having unmet ADL/IADL needs.

**Method:**

In 2021‐2022 the caregivers of 741 Veterans with dementia completed round one of the HERO CARE survey, a prospective longitudinal panel design survey administered to caregivers and VHA‐enrolled Veterans across five VHA sites, with oversampling of Veterans with higher predicted risk of long‐term institutionalization. 314 of these surveys had sufficient response data for inclusion in the current analysis. Multiple linear regression was used to regress the outcome of combined total score of unmet ADL/IADL needs (minimum = 0, maximum = 15, mean = 4.86, SD = 4.92) on specific demographic characteristics and comorbidities (using Hierarchical Condition Categories) across five models. The model of best fit was identified by AIC.

**Result:**

Non‐white/non‐Hispanic race‐ethnicity (β = 3.14, se = 0.64) and having Parkinson’s or Huntington’s diseases (β = 2.718, se = 0.870) were associated with having more unmet ADL/IADL needs at the 5% significance level. Having major depressive, bipolar, and/or paranoid disorders (β = ‐ 1.395, se = 0.656) was associated with having less unmet ADL/IADL needs at the 5% significance level. When controlling for the other predictors, having non‐white/non‐Hispanic race‐ethnicity was associated with an average increase of 3.25 unmet ADLs/IADLs, having Parkinson’s or Huntington’s Disease was associated with an average increase of 2.72 unmet ADLs/IADLs and having major depressive, bipolar, and/or paranoid disorders was associated with a 1.40 decrease in unmet ADLs/IADLs.

**Conclusion:**

The Veterans Affairs Healthcare System (VAHS) may have characteristics that support the provision of ADL/IADL assistance for patients who have dementia in conjunction with major depressive, bipolar and/or paranoid disorders. Increased attention to ADL/IADL supports among VAHS patients who have dementia and non‐white/non‐Hispanic race/ethnicity and/or Parkinson’s or Huntington’s diseases may support Veteran health, wellbeing, and aging in place.

